# Ultrasound predicts skeletal muscle fat infiltration in healthy middle‐aged and young adults: Validation against MRI

**DOI:** 10.1113/EP092865

**Published:** 2026-03-04

**Authors:** RK Holsgrove‐West, PM Słowiński, E Svensen, C Koscien, J Fulford, R Revuelta Iniesta, BT Wall, FB Stephens

**Affiliations:** ^1^ Public Health and Sport Sciences, Medical School University of Exeter Exeter UK; ^2^ Department of Mathematics and Statistics, Faculty of Environment, Science and Economy University of Exeter Exeter UK; ^3^ Medical Imaging Department, Faculty of Health and Life Sciences University of Exeter Exeter UK

**Keywords:** ageing, echointensity, fat infiltration, magnetic resonance imaging, muscle, ultrasound

## Abstract

This study developed an ultrasound prediction equation for measuring fat fraction in lower limb muscles of healthy middle‐aged and young participants and applied it in a randomised controlled trial (RCT) to assess the relationship between fat fraction and strength in exercising and non‐exercising females. Twenty‐eight participants were recruited into <40 years (10m, 5f, age 28.9 ± 5.3 years, body mass index (BMI) 23.6 ± 2.3 kg m^−2^) and >50 years (8m, 5f, age 60.4 ± 5.7 years, BMI 24.8 ± 3.6 kg m^−2^) groups. T1‐weighted magnetic resonance imaging (MRI)‐Dixon fat fraction in gastrocnemius medialis (GM) and vastus lateralis (VL) and ^1^H‐magnetic resonance spectroscopy for intramyocellular (IMCL) and extramyocellular (EMCL) lipid content in GM was used to validate ultrasound measures of muscle, subcutaneous fat thickness (SFT) and echointensity to generate a multivariable model of fat infiltration. For the RCT, 72 females aged 40–60 years were recruited to either a low impact resistance exercise (4–5 sessions/week for 12 weeks) or a control group, and muscle strength was tested using isokinetic dynamometry. Multiple linear regression models for ultrasound measurement of fat fraction were validated for GM (*r* ^2^ = 0.71) and VL (*r* ^2^ = 0.91). SFT was the dominant variable in the older group in GM (77%) and VL (92%), but of near equal proportion, with echointensity, in GM (52%) and VL (51%) in the younger group. Strength was negatively correlated with fat fraction (*r* = −0.44). Strength increased in the exercising group, but fat fraction remained unchanged. A multivariable model using ultrasound measurements of SFT, muscle thickness, echointensity and age demonstrates that ultrasound is capable of predicting lower limb muscle fat infiltration.

## Abstract (highlights)

- **What is the central question of this study?**
  Can a hand‐held ultrasound device be used to measure fat fraction in lower limb muscles of healthy middle‐aged and young participants?
- **What is the main finding and its importance?**
  Yes, ultrasound can be used to predict fat fraction using a multivariable model using subcutaneous fat thickness, muscle thickness, echointensity and age. Echointensity used alone is shown to be a poor predictor of intramuscular fat content and is more likely to be the output of all muscle parameters, rather than just fat content. Hand‐held ultrasound has potential as a user‐friendly, bedside clinical tool which is cheaper and more easily accessible than MRI.

## 1 INTRODUCTION

Declines in muscle quantity and quality have been associated with functional decline in older adults (Correa‐de‐Araujo et al., 2017; Crane et al., 2010; Visser et al., 2005). After 30 years of age, muscle mass decreases by 3–8% per decade (Melton et al., 2000), and is considered the primary determinant of age‐related declines in strength (Frontera et al., 1991). Losses in strength, however, appear to exceed losses in muscle quantity, and there is increasing evidence that poor muscle quality, often associated with the infiltration of fat, may have a greater impact on functional strength than quantity alone (Fragala et al., 2015). Developing a tool capable of measuring and predicting age‐related changes, in both muscle fat infiltration and quantity, would assist clinicians and researchers in understanding age‐related changes to interventions and treatments designed to promote function into old age.

Magnetic resonance imaging (MRI) using histogram thresholding techniques from T1‐weighted images, with or without Dixon‐based sequencing, is the current gold standard for measuring intramuscular fat percentage, with proton magnetic resonance spectroscopy (^1^H‐MRS) capable of further separating intramyocellular (IMCL) and extramyocellular (EMCL) lipid. However, both are expensive and often inaccessible for standard clinical practice. Ultrasound is a more affordable and accessible imaging tool, which has been validated against MRI as a robust measure of muscle mass (Sanada et al., 2006), cross‐sectional area (CSA; Franchi et al., 2018) and thickness (Mechelli et al., 2019, Worsley et al., 2014). Moreover, ultrasound echointensity has previously been used as a measure of muscle quality in both healthcare, in humans with muscular dystrophy (Pillen et al., 2011), and in agriculture, to assess intramuscular fat content of livestock (Fabbri et al., 2021; Indurain et al., 2009). These are based on muscle quality being defined as the amount of fat in a muscle, rather than its truest definition of strength or force per CSA or mass, and on the assumption that fat has higher reflective properties than lean muscle and that an increase in intramuscular fat will lead to a concomitant and proportional increase in echointensity. Echointensity, however, is an acoustic‐based technique, influenced by a variety of factors other than fat, which affects its reliability and validity. Muscle quality, in this study, has been defined as strength per unit body mass.

Early ultrasound models seeking to predict fat infiltration suggest correcting for interference from subcutaneous fat thickness (SFT; Reimers et al., 1993). Indeed, moderate to strong correlations were found between models of SFT‐*corrected* ultrasound echointensity and both T1‐weighted MRI fat signal intensity (Young et al., 2015) and total intramuscular fat using ^1^H‐MRS (Piponnier et al., 2023), in several lower limb muscles of young participants. However, intramuscular fat (EMCL) of the vastus lateralis (VL) and biceps femoris, measured using both T1‐weighted MRI and ^1^H‐MRS, was strongly correlated with *uncorrected* ultrasound echointensity in a group of young and older participants (Akima et al., 2016), suggesting that further refinement is necessary. Indeed, while these models propose to use echointensity as a surrogate measure for fat infiltration they do not take into account thicknesses of different muscles or age, which are likely to affect the reflective properties of the muscle.

This study, therefore, aimed to develop a prediction equation for ultrasound echointensity that is capable of measuring fat infiltration in lower limb muscles, by including SFT, muscle thickness and age as covariates and, for the first time, comparing to high resolution T1‐weighted MRI with Dixon sequencing and ^1^H‐MRS measures of IMCL and EMCL. To assist in equation development, the relationship between SFT and echointensity was investigated in an *ex vivo* system (pork tissue), to either validate or revise the derivation of the SFT correction factor from Young et al. (2015), and subsequently validated in a randomised controlled trial (RCT) exercise intervention designed to improve muscle function in middle aged females.

## 2 METHODS

### 2.1 Study 1: Comparison of ultrasound prediction equation against MRI and ^1^H‐MRS

#### 2.1.1 Participants

Twenty‐eight participants between the ages 21 of and 69 years were recruited into two groups: younger <40 years (10 male, 5 female) and older >50 years (8 male, 5 female). Exclusion criteria were the standard MRI exclusions (Ghadimi, 2023).

The study was approved by the NHS Healthcare Research Authority (Ref: 22/HRA/2343) and was conducted in accordance with the 1964 *Declaration of Helsinki* (last modified in 2013) and registered at clinicaltrials.gov (NCT05729880). Data were collected from August to November 2022.

#### 2.1.2 Study design

Ultrasound, MRI and ^1^H‐MRS testing were completed sequentially in a single 1 h session. On arrival, participants rested in the long sitting position for 20 min. Sites were carefully marked, ultrasound images were taken and a fish oil capsule was taped to the site where the ultrasound image was taken. This enabled precise matching of the ultrasound site to MR image and aided in defining the MRS voxel location. Participants were then taken to the MR scanner in the adjacent room. Ultrasound body positions were replicated in the scanner for each muscle.

#### 2.1.3 Ultrasound protocol

##### 2.1.3.1 Muscle thickness, SFT and echointensity

A portable, handheld, wireless B‐mode 7.7 Hz linear transducer, 96% brightness, 6 cm depth, ultrasound device was used for all measurements (VScan Air, GE Healthcare, Chalfont St Giles, UK). In accordance with suggested standardised protocols (Fujiwara et al., 2010), GM measurements were taken with the subject lying prone, one‐third of the distance proximal to the right medial knee joint line and medial malleolus. VL measurements were taken with the subject lying supine and measured as 50% from anterior superior iliac spine to the lateral joint line of the right knee. Once the site had been clearly marked, generous amounts of ultrasound gel was applied to the probe, which was first held vertically in the transverse plane to allow clear detection of the subcutaneous fat layer and superficial and deep aponeuroses of the muscles. The probe was then rotated 90 degrees into the longitudinal plane. Three images of each were taken, downloaded from the GE VScan app, saved as a JPEG, analysed using ImageJ (version 1.53e) and averaged.

**FIGURE 1:**
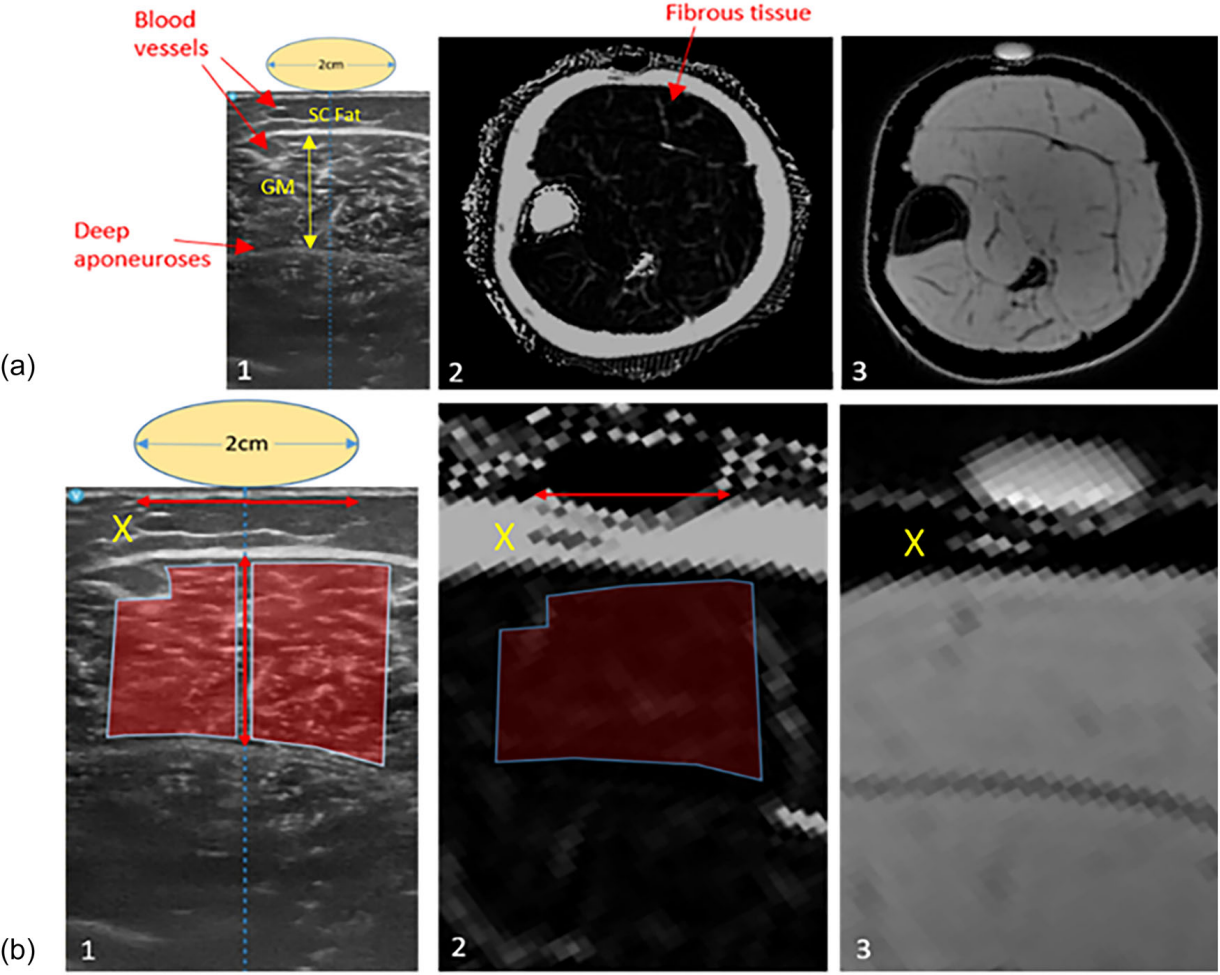
(a) Gastrocnemius medialis (GM) ultrasound anatomical identifiers, thickness and subcutaneous (SC) fat layer (1), MRI fat fraction map (2), and MRI water fraction map (3). (b) The respective magnified locations for measurements of ultrasound echointensity (1), fat fraction percentage (2) and water image (3) for reference. X, matching anatomical landmark.

Muscle thickness and SFT were measured from the transverse image using the line function and calibrated according to Cronin (2022). For both GM and VL, thickness was measured as the distance between superficial and deep aponeurosis, and subcutaneous fat was measured as the skin line to the superficial aponeuroses (Figure 1a1–3). Owing to the shallow pennation angle of VL, pennation angle was measured in GM only as the angle between fascicle and deep aponeuroses.

Echointensity was measured using the greyscale histogram function (0–255 AU), and the freehand rhomboid tool to outline the muscle region of interest. Owing to the presence of the centreline on the ultrasound image (which matched the centre of the fish oil capsule) two regions of interest were drawn either side and averaged. (Figure 1b1–3).

#### 2.1.4 MRI protocol

All MR data acquisition was performed on a Siemens 3.0 T MR scanner (MAGNETOM Prisma, Siemens Healthineers, Erlangen, Germany). Two separate scanning acquisitions were acquired of the gastrocnemius and VL muscles with repositioning between each with the order of measurements randomised.

For the calf muscle the participant lay prone upon the scanner bed with their calf placed in the centre of a 15‐element knee coil and the foot supported such that the calf was not compressed within the coil. After initial scout images were taken to confirm appropriate positioning, a high‐resolution anatomical sequence was employed to generate a sequential series of transverse slices. The slice that contained the centre of the fish oil capsule was located and used as the reference slice for subsequent measurements. A T1–VIBE–Dixon sequence was subsequently run centred on this slice to generate a transverse set of images. The sequence employed a 320 mm field of view resulting in an in‐plane resolution of 1 × 1 mm and a slice thickness of 1.5 mm. Images were acquired with six echo times (TE) ranging from 1.34 to 9.99 ms and a repetition time (TR) of 11.80 ms. Subsequently, fat and water fraction maps were generated for each slice, which were corrected for T2 relaxation effects and represented the percentage of signal that could be attributed to each component on a voxel‐by‐voxel basis.

At the completion of imaging ^1^H spectroscopy was undertaken. A single voxel PRESS technique was employed with a voxel 15 × 15 mm in plane and 20 mm in the foot–head direction. The voxel was positioned on the reference slice and positioned such that it was located adjacent to the fish oil capsule, but not overlying subcutaneous fat. The reference slice was the central slice for the voxel in the foot–head direction. Two separate spectra were acquired, one without water suppression and one with, but all other parameters kept constant. Twenty‐four separate acquisitions were averaged for each spectrum with a TR = 3000 ms and a TE of 30 ms. Prior to the first of the two spectral acquisitions, 90° pulse optimisation was performed and manual shimming was undertaken to ensure a full width at half‐maximum of less than 20 Hz. All optimisation settings were kept constant for the second acquisition.

On completion of the calf measurements the participant was repositioned such that they lay supine with a wrap‐around body coil positioned around the upper leg and centred at the site of the fish oil capsule. After scout and anatomical images were acquired, the slice containing the fish oil capsule was identified in the same way as for the calf measurements and again a T1–VIBE–Dixon sequence was run centred on this slice to generate a transverse set of images. The sequence employed a 450 mm field of view resulting in an in‐plane resolution of 1.4 × 1.4 mm and a slice thickness of 1.5 mm. Images were acquired with six TE ranging from 1.34 to 9.39 ms and a TR of 11.10 ms. Fat fraction maps were then generated in the same way as for the calf.

#### 2.1.5 MRI analysis

MR images of the fat fraction map for both calf and thigh were analysed using ImageJ (version 1.53e). Unlike other imaging techniques Dixon sequencing provides an image with precise fat content (Bray et al., 2018). Each fat fraction slice was visually scrutinised, and the slice representing the centre of the fish oil was chosen for analysis. The muscle region of interest was drawn, and the histogram of voxel intensity obtained for fat fraction percentage. For ultrasound analysis, simultaneously visualising the MR image with ultrasound image, it was possible to identify muscle borders and anatomical landmarks to optimise matching. The muscle region of interest was drawn, and the mean histogram intensity was chosen as the echointensity. Both were repeated three times and averaged.

#### 2.1.6 MRS analysis

All spectra were transferred into the java‐based MR user interface jMRUI (version 5.2) for processing. For the water suppressed spectrum, the remaining water peak was further suppressed and the areas of the creatine, IMCL and EMCL peaks calculated using the AMARES (Advanced Method for Accurate, Robust and Efficient Spectral fitting) fitting algorithm (Vanhamme et al., 1997) with the areas of the IMCL‐CH_2_ peak at 1.3 ppm and EMCL‐CH_2_ peak at 1.5 ppm used for subsequent metabolite quantification. The unsuppressed water spectrum was used for water peak quantification. In order to determine absolute concentrations of IMCL and EMCL, peak areas were compared with the water peak area, correcting for T1 and T2 relaxation (Valaparla et al., 2015), relative numbers of protons within the respective molecules and assuming a water concentration within muscle of 40 mmol/g wet weight (Wang et al., 2009).

### 2.2 Study 2: Investigation of the relationship between subcutaneous fat and echointensity in an *ex vivo* system (pork tissue)

To investigate the relationship between SFT and echointensity a separate small study was performed using pork.

Repeated measures on five pork loins, from the same pig, were chosen for uniformity of the subcutaneous fat layer. The loin was laid flat and ultrasound gel was applied. Using settings identical to Study 1, SFT, muscle thickness and echointensity were measured. A thin layer of subcutaneous fat was systematically sliced off in as close to a millimetre slice as possible, and the loin was re‐measured. This continued until no fat remained and a measurement of pure pork muscle was obtained. The mean of three images was obtained at each stage.

### 2.3 Study 3: Association between ultrasound predicted fat fraction and strength in women aged 40–60 years, undertaking a 12‐week low impact resistance exercise programme – a RCT

A large, RCT (FemME Study) to assess the effectiveness of a 12‐week resistance exercise training programme on muscle function in women aged 40–60 years was undertaken in our research group. This study was approved by the NHS Healthcare Research Authority (Ref: 22/YH/0235) and was conducted in accordance with the 1964 *Declaration of Helsinki* (last modified in 2013). Written informed consent from all volunteers was obtained prior to participation in the study. Data were collected from May 2022 to November 2023. The full protocol can be found on clinicaltrials.gov (NCT05397418).

In brief, 72 moderately active females aged 40–60 years, body mass index (BMI) <30 kg m^−2^ and >18.5 kg m^−2^ were randomised into either the control or the exercise group. Exercise consisted of 12 weeks of a pre‐set programme of low impact resistance exercise classes 4–5 times per week while the control group continued habitual life. Pre–post ultrasound measures of muscle thickness, SFT and echointensity were taken and analysed in a manner identical to that described in Study 1, in the rectus femoris, in the transverse plane, at 50% distance between ASIS and superior pole of patella. Fat fraction was predicted using the equation generated from Study 1. Pre–post strength was measured as peak torque of hip flexion using an isokinetic dynamometer (Biodex System 3, cat. no. 830‐200, Shirley, NY, USA).

### 2.4 Statistical analysis

Ultrasound muscle thicknesses were validated against MRI using Pearson's correlation coefficient and Bland–Altman plots and correlated with MRI CSA. To establish whether the corresponding ultrasound segment on the MRI fat fraction was representative of the whole, CSA fat fraction was compared with US segment fat fraction and agreement was analysed using Bland–Altman plots (1986). Echointensity was correlated with MRI fat fraction (GM and VL), and EMCL and IMCL (GM only), using Pearson's correlation coefficient to provide anatomical insight. To understand the relationship between SFT and echointensity in the pork model system, a best fit negative exponential was applied. SFT was then log transformed and the gradient used to establish a correction factor (corrected echointensity = gradient × log (SFT) + echointensity).

To develop the prediction equation of muscle fat infiltration, simple linear regression was performed to examine the relationships between MRI fat fraction and ultrasound echointensity, SFT, muscle thickness and age. Including the significant variables, a multiple linear regression model was produced, using MRI fat fraction as a dependent variable and ultrasound echointensity, SFT, muscle thickness and age as independent variables. To understand the model, variance‐based importance measures (VIMs, Grömping, 2007) were used to decompose the full *r* ^2^ into contributions from the regressors and rank the independent variables in order of contribution to the variance. The Shapiro–Wilk test for normality was performed and variable inflation factors were checked for multicolinearity with all <1.5 (i.e., not co‐linear). For agreement with fat fraction, the new equation was analysed alongside current models (echointensity alone; SFT alone and echointensity adjusted for SFT) using Bland–Altman plots. Coefficients of determination (*r* ^2^) were then compared between models. For Study 3, a two‐way ANOVA was used to determine time × group interaction for fat fraction and strength changes in exercising and control groups. Simple linear regression was used to assess the relationships between fat fraction and strength. Statistical analyses were performed using GraphPad Prism 9 (GraphPad Software, Boston, MA, USA). Data are presented as means ± SD. Significance was accepted when *P* < 0.05.

## 3 RESULTS

Participant characteristics are shown in Table 1.

**TABLE 1:** Measures of ultrasound, MRI and ^1^H‐MRS parameters in young and middle‐aged participants.

	Young (<40 years) *n* = 15	Middle‐aged (>50 years) *n* = 13
Age (years)	28.9 (5.3)	60.4 ^1^ (5.7)
Height (cm)	174.8 (8.1)	173.9 (8.7)
Weight (kg)	72.0 (9.2)	75.3 (13.8)
BMI (kg m^−2^)	23.6 (2.3)	24.8 (3.6)
GM EI (AU)	82.5 (7.3)	92.3 ^1^ (13.3)
GM SC Fat (cm)	0.5 (0.1)	0.7 (0.4)
GM IMCL (mmol kg^−1^ ww)	11.97 (10.6)	7.57 (6.19)
GM EMCL (mmol kg^−1^ ww)	16.82 (14.28)	35.54 ^1^ (21.9)
VL Tm (cm)	2.4 (0.4)	2.1 ^1^ (0.3)
VL CSA (cm^2^)	22.1 (5.0)	17.5 (7.6)
VL MRI fat fraction – total CSA (%)	2.1 (0.7)^4^	4.7 ^1,4^ (2.3)
VL MRI fat fraction – US segment (%)	2.1 (1.0)	3.8 ^1^ (2.2)
VL EI (AU)	72.7 (9.5)	84.9 ^1^ (15.8)
VL SC fat (cm)	0.7 (0.4)	0.9 (1.0)

Data are mean (SD). ^1^Significant diff middle‐aged vs. young, *P *< 0.05. ^2–4^Pearson's correlation between MRI fat fraction %, CSA and corresponding US segment in ^2^GM young, *r* = 0.95, *P* < 0.0001 and ^3^GM middle‐aged *r* = 0.92, *P* < 0.0001, ^4^VL young *r* = 0.90, *P *< 0.0001, middle‐aged *r* = 0.90, *P *< 0.0001. Abbreviations: BMI, body mass index; CSA, cross‐sectional area; EI, echointensity; EMCL, extramyocellular lipid; GM, gastrocnemius medialis; IMCL, intramyocellular lipid; SFT, subcutaneous fat thickness; Tm, muscle thickness; VL, vastus lateralis.

### 3.1 Ultrasound fat infiltration prediction model

Multiple linear regression models for fat fraction (FF), incorporating significant variables for both GM (equation 1, *r* ^2^ = 0.71, adj *r* ^2^ = 0.68, SFT *P *< 0.0001, echointensity *P* = 0.0014, Tm *P* = 0.0427) and VL (equation 2, *r* ^2^ = 0.91, adj *r* ^2^ = 0.89, SFT *P *< 0.0001, echointensity *P *< 0.0001, Tm *P* = 0.0091, age *P* = 0.0323) are:

(1) $$\mathrm{GM}\;\mathrm{FF}\;\%\;=\left(\mathrm{SFT}\times 5.811\right)+\left(\mathrm{EI}\times 0.0877\right)+\left(\mathrm{Tm}\times 2.019\right)-10.27$$

(2) $$\begin{array}{ccc} \mathrm{VL}\;\mathrm{FF}\;\%\; & = & \left(\mathrm{SFT}\times 2.451\right)+\left(\mathrm{EI}\times 0.05512\right)+\left(\mathrm{Tm}\times 1.170\right)\\ & & +\;\left(\mathrm{Age}\times 0.01878\right)-6.615 \end{array}$$

where SFT is subcutaneous fat thickness, EI is echointensity and Tm is muscle thickness. To understand how much each of the independent variables contributes to the model in terms of the explained variance, the variable importance measures (VIMs; Grömping, 2007) are shown in Table 2. SFT is consistently the major contributor influencing fat fraction and is more dominant in older participants.

**TABLE 2:** Variable importance measures for regressors involved in multiple linear regression fat fraction prediction model.

VIMs	GM			VL		
	Total	Young, <40 years	Middle‐aged, >50 years	Total	Young, <40 years	Middle‐aged, >50 years
*r* ^2^ (adj *r* ^2^)	0.71 (0.68)			0.91 (0.89)		
SFT	0.55	0.28	0.55	0.71	0.42	0.84
EI	0.11	0.26	0.08	0.13	0.30	0.07
Tm	0.04	0.00	0.07	0.03	0.11	0.00
Age	0.02^*^	0.00	0.00	0.03	—	—
Total	0.70	0.54	0.70	0.90	0.83	0.91

Adj *r* ^2^ accounts for multivariables. ^*^Not significant, not included in regression equation. SFT, subcutaneous fat thickness.

**FIGURE 2:**
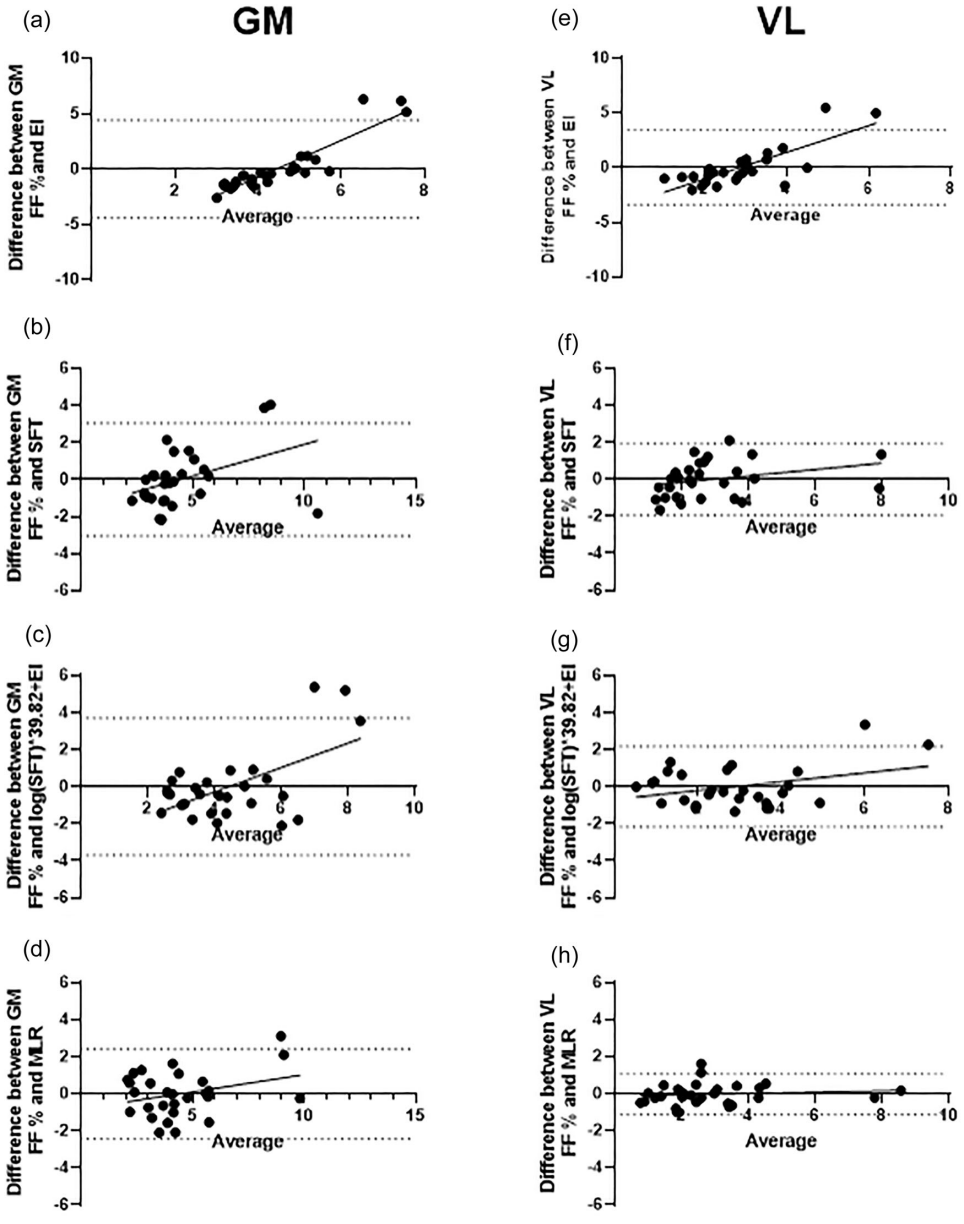
Bland–Altman analyses measuring agreement between MRI fat fraction percentage and fat fraction percentage predicted by the four models for GM (a–d) and VL (e–h).

Bland–Altman analyses using the raw, untransformed data, measuring agreement between MRI fat fraction percentage and the fat fraction percentage predicted from the four models for both VL and GM are shown in Figure 2 and their values in table 3. Echointensity and SFT prediction methods demonstrated heteroscedascity with the funnel shape plots showing increased difference with higher fat fractions (Figure 2a, b, e, f) whereas the multivariable model (Figure 2d, h) shows no proportional bias. Regression analyses (Table 2) demonstrated MLR had the highest *r* ^2^ value.

**TABLE 3:** Bland–Altman analyses for agreement between MRI fat fraction and models of fat fraction prediction.

	Echo intensity	SFT	Present study EI + SFT	Multivariable model
GM fat prediction model				
Equation	*y* = (0.0444 × EI) + 0.575	*y* = (5.4480 × SFT) + 1.388	*y* = (0.0968 × adj EI) – 2.8373	(SFT × 5.811) + (EI × 0.0877) + (Tm × 2.019) – 10.27
*r* ^2^	0.05	0.55	0.34	0.71 (adjusted 0.68)
*P* value	0.2622	< 0.0001	0.0012	< 0.0001
Bland–Altman bias	−0.002	0.001	−0.009	0.003
SD	2.26	1.55	1.89	1.24
95% LoA upper, lower	4.43, −4.43	3.04, −3.04	3.7, −3.7	2.432, −2.426
VL fat prediction model				
Equation	*y* = (0.0511 × EI) – 1.1267)	*y* = (2.2086 × SFT) + 1.2736	*y* = (0.0781 × adj EI) – 2.3414	(SFT × 2.451 + (EI × 0.0551) + (Tm × 1.170) + (Age × 0.0188) – 6.615
*r* ^2^	0.13	0.72	0.65	0.91 (adjusted 0.89)
*P* value	0.0545	< 0.0001	< 0.0001	< 0.0001
Bland–Altman bias	0.001	0.0001	−0.1096	−0.04
SD	4.74	1.16	1.117	0.57
95% LoA upper, lower	9.29, −9.29	2.3, −2.3	2.1, −2.3	1.07, −1.15

Abbreviations: EI, echointensity; GM, gastrocnemius medialis; SFT, subcutaneous fat thickness; VL, vastus lateralis.

### 3.2 Muscle quantitative measures

MRI fat fraction and raw echointensity values were significantly lower in younger compared with older participants in both GM and VL (Table 1), with greater variance in the older population. Whole group mean MRI fat fraction measured from CSA and fat fraction measured at the corresponding ultrasound segment were similar in both the GM (4.2 ± 2.1% and 4.4 ± 2.3%, Bland–Altman 0.29 ± 0.75, 95% limits of agreement ((LoA) −1.2 to 1.8) and VL (3.3 ± 2.1% and 2.9 ± 1.9%, Bland–Altman 0.40 ± 0.89, 95% LoA −2.1 to 1.3). Median VL fat fraction from MRI was 2.6%, lower 95% 2.2, upper 95% CI 3.6 and GM 3.8% lower 95% 3.5, upper 95% CI 5.3.

Mean ultrasound and MRI muscle thickness measurements were similar for GM (1.85 ± 0.3 and 1.87 ± 0.3, Bland–Altman −0.04 ± 0.13, 95% LoA −0.29 to 0.22, *r* = 0.90, *P* < 0.0001) and VL (2.28 ± 0.40 and 2.22 ± 0.36, Bland–Altman 0.03 ± 0.22, 95% LoA −0.4 to 0.5, *r* = 0.83, *P* < 0.0001). Ultrasound muscle thickness was strongly correlated with MRI CSA for both GM (*r* = 0.81, *P *< 0.0001) and VL (*r* = 0.76, *P *< 0.0001). Muscle thickness was not different between young and older groups for GM (*P* = 0.9838) and VL (*P* = 0.0527) and ultrasound thickness measurement variability was low for GM (CV 4.7%) and VL (CV 2.2%). Pennation angle was negatively correlated with echointensity (*r* = −0.50, *r* ^2^ = 0.21, *P* = 0.0133) and weakly correlated with age (*r* = 0.37, *P* = 0.0493, mean young 34.6 ± 6.7° and middle‐aged 30.5 ± 5.0°).

### 3.3 Muscle quality measures

**FIGURE 3:**
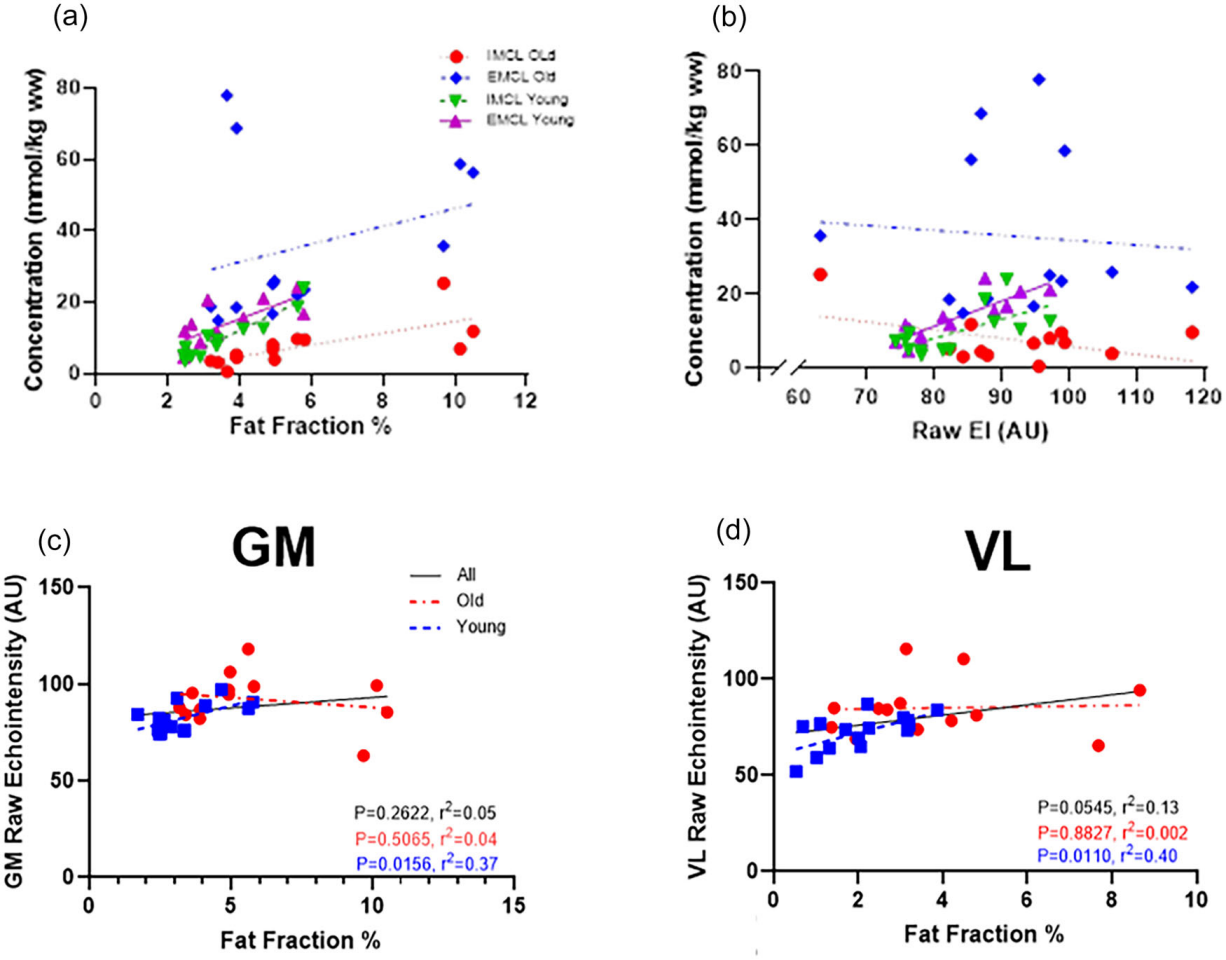
(a, b) Correlation between concentrations of intramyocellular lipid (IMCL) and extramyocellular lipid (EMCL) in the gastrocnemius medialis muscle with (a) fat fraction and (b) echointensity. (c, d) Linear regression of fat fraction and echointensity in (c) gastrocnemius medialis (GM) and (d) vastus lateralis (VL) in all, young and middle‐aged participants.

**FIGURE 4:**
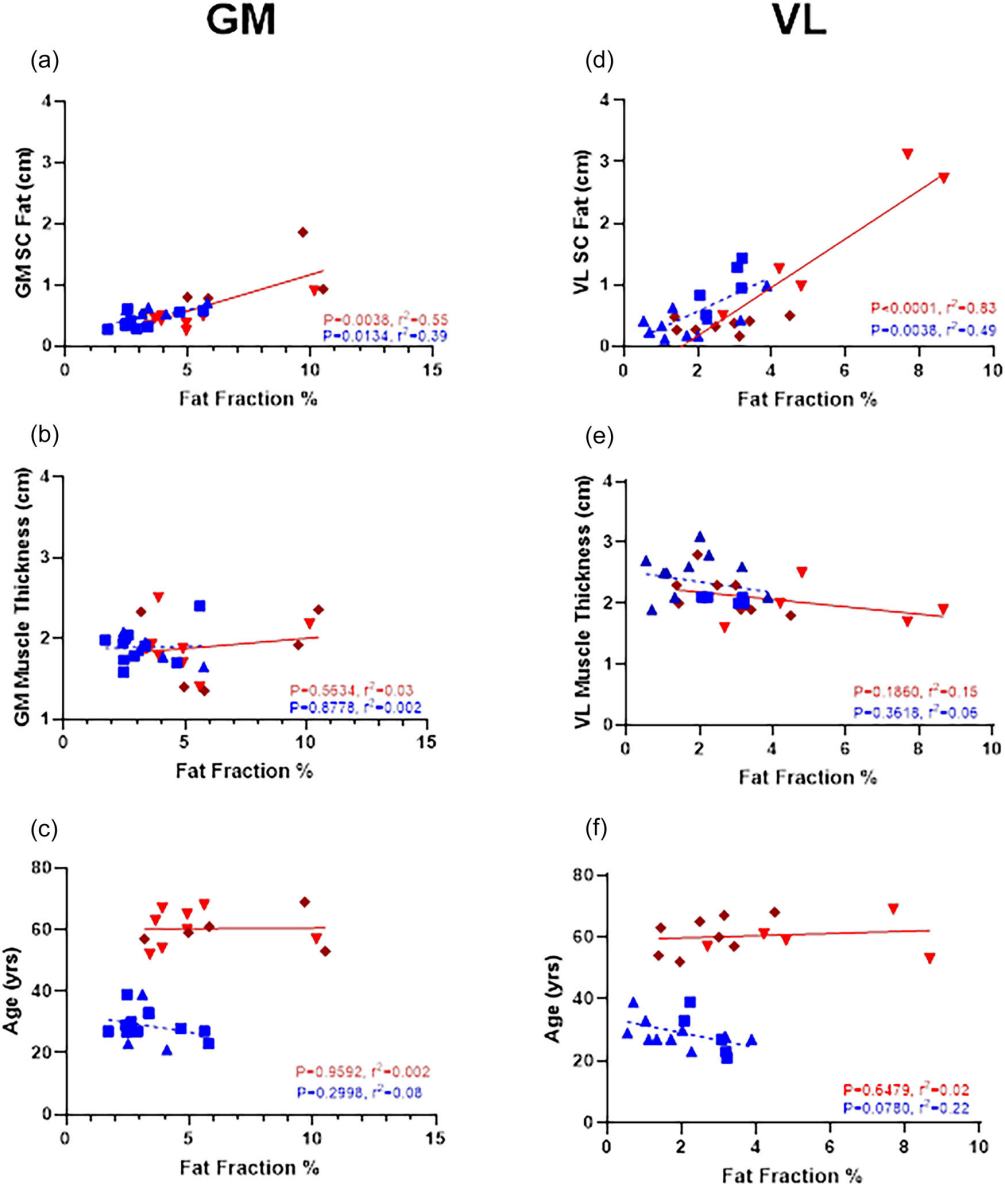
Young (blue) and middle‐aged (red) linear regression of gastrocnemius medialis (GM, left column a–c) and vastus lateralis (VL, right column d–f) between fat fraction and subcutaneous fat thickness (SFT) (a and d), muscle thickness (b and e) and age (c and f).

MRI fat fraction was correlated with both IMCL and EMCL (*r* = 0.54, *P* = 0.0045 and *r* = 0.53, *P* = 0.0050, respectively) for the whole group, whereas echointensity was not (*r* = 0.02, *P* = 0.5175 and *r* = 0.07, *P* = 0.1779, respectively). When separating for age, MRI fat fraction (Figure 3a) was correlated with IMCL in young (*r* = 0.95, *P *< 0.0001) and middle‐aged (*r* = 0.70, *P* = 0.0077), and EMCL in young (*r* = 0.87, *P* = 0.0030) but not middle‐aged (*r* = 0.30, *P* = 0.3205). Echointensity (Figure 3b) was correlated with IMCL and EMCL in the young groups only (*r* = 0.64, *P* = 0.0181 and *r* = 0.85, *P* = 0.0002, respectively). Correlations between fat fraction and ultrasound measures of echointensity, SFT, muscle thickness and age for GM and VL are shown in Figure 4.

### 3.4 Relationship between SFT and echointensity in a pork *ex vivo* system

**FIGURE 5:**
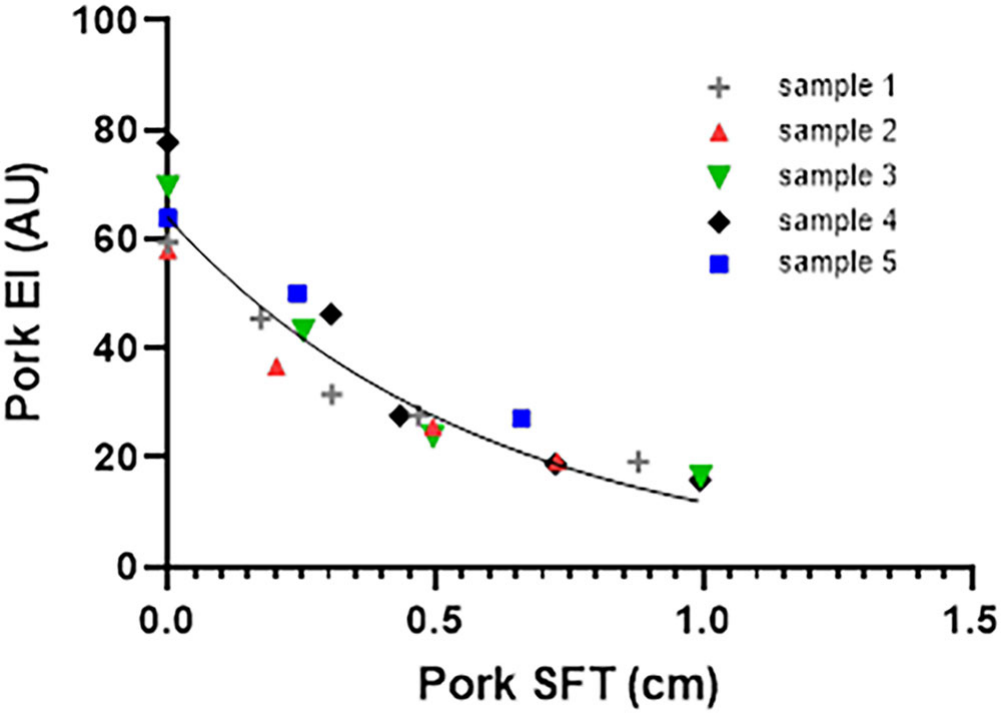
Relationship of pork subcutaneous fat thickness (SFT) and echointensity (EI) with data combined from 5 samples. *r* ^2^ = 0.90, *y* = 64.0 × exp^(−1.69 × SFT)^, semilog transformation of data *y* = −39.82 × log(SFT) + 15.51.

There was a negative exponential relationship (*r* ^2^ = 0.89) between ultrasound echointensity values of the pork and SFT (Figure 5). When applying this relationship to the echointensity data collected on human participants, using the gradient of −39.82, so that for every 1 cm of log(SFT), the echointensity decreases by −39.82 AU, the correlation between raw and adjusted echointensity and fat fraction changed from non‐significant to significant in both GM (*P* = 0.2622 to *P* = 0.0012) and VL (*P* = 0.0545 to *P *< 0.0001). This demonstrates how the significance found between adjusted echointensity and fat fraction is due to the strong relationship with SFT.

### 3.5 Fat fraction and strength

**FIGURE 6:**
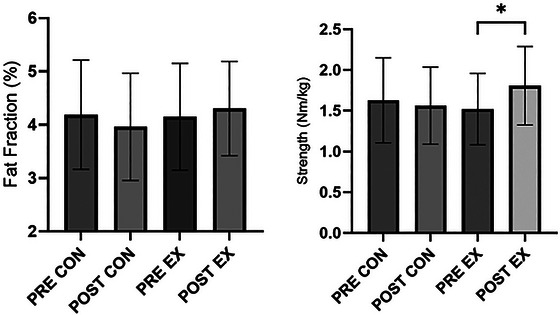
Pre–post fat fraction and strength measurements for control and exercise groups. Fat fraction did not change pre–post in the control and exercise groups whereas post‐exercise strength was significantly higher than pre‐exercise strength (*P* = 0.0255).

Figure 6 shows that strength was significantly increased post‐exercise intervention (1.52 ± 0.44 N m/kg to 1.81 ± 0.48 N m/kg, *P *< 0.01) whereas it did not change in the control group (1.63 ± 0.52 N m/kg to 1.56 ± 0.47 N m/kg, *P* = 0.24). Fat fraction did not change pre–post in the exercise (4.15 ± 1.00% and 4.31 ± 0.88%, respectively, *P* = 0.12) or control group (4.19 ± 1.02% and 3.96 ± 1.00%, respectively, *P* = 0.07). Strength showed moderate negative correlation with fat fraction pre‐ and post‐ in control (*r* = −0.40, *P* = 0.0484 and *r* = −0.44, *P* = 0.0278, respectively) and exercise (*r* = −0.34, *P* = 0.0223 and r = −0.44, *P* = 0.0024, respectively) groups. A two‐way ANOVA showed a time–group interaction (*F*_(1,68) _= 5.817, *P* = 0.0186). Muscle thickness was greater post‐exercise (*P* = 0.0173) whereas SFT and echointensity did not change (0.7197 and 0.8011, respectively). There was no difference in baseline values between control and exercise groups for fat fraction (4.19 ± 1.02% and 4.15 ± 1.00%, *P* = 0.54) or strength (1.63 ± 0.52 N m/kg and 1.52 ± 0.44 N m/kg, *P* = 0.30).

## 4 DISCUSSION

We present two multivariable ultrasound equations capable of predicting fat fraction for GM and VL that are comparable to gold standard MRI for fat fraction and that outperform existing models and correlate with muscle strength in middle aged females. Whilst all models for both GM and VL showed minimal constant bias, when compared with MRI fat fraction, the limits of agreement were lowest in the multivariable models, and with a greater coefficient of determination. This means that the mean fat fraction was similar to the MRI value with a larger portion (VL 89%, GM 68%) of the variability explained by the model and the real fat fraction value sitting within a narrower limit. Mean VL fat fraction from the multivariable model was 3.3% with limits of agreement ±1.1% suggesting the true value lies between 2.2% and 4.4%. This could be of use clinically to assess change over time, but not yet diagnostically. For the EI‐only model, limits of agreement were ±9.29%, clearly of little clinical or diagnostic value.

The EI‐only and the SFT‐only models showed proportional bias. This means that the difference between predicted value and MRI FF percentage increased as fat fraction increased. Physiologically, those participants with greater SFT could indeed have more muscular fat, but, as shown in the pork model here, the ultrasound signal is attenuated with greater SFT. EI and SFT are not independent variables and therefore are not appropriate models to use. The multivariable model takes into account these variables plus muscle thickness and age and demonstrates little proportional bias. Whilst the MLR model outperformed other models, there were a large number of data points clustered around the mean with only a few with higher fat fractions. A wider range of data points, involving participants with greater fat fraction would refine the model further to determine minimal clinical differences, and we suggest the current model is valid only for fat fractions between 2% and 6%. To our knowledge, these are the first equations to include muscle thickness and age, which were lesser but significant contributors, and markedly improved the utility of ultrasound for measuring fat fraction and muscle quality.

Proportioning and ranking the variables highlighted a clear difference between young and older participants. The young group had a more even contribution between SFT and echointensity compared with the older group, who were strongly dominated by SFT (in VL, 84 of the 91% can be explained by SFT alone). This finding can be explained, mathematically, by the greater variation in echointensity in the older group. Physiologically, it is less clear why echointensity is more variable in older than younger participants, but it could be related to differences in tissue homogeneity.

Echointensity is the mean greyscale value of sounds reflected off the interface between two tissues with different acoustical impedance. Homogeneous subcutaneous fat is hypoechoic, and we have shown that increasing thickness attenuates the echointensity distal to this. Homogeneous muscle tissue is also hypoechoic but is separated by hyperechoic sheets of perimysium surrounding the fascicles shown as the speckled image on the transverse plane and the pennate structure on the longitudinal plane. Lowering pennation angle, as might happen with decreasing muscle thickness and increasing age (Narici et al., 2003), and shown here in the VL older group, increases angle of incidence and reflection and thus gives greater echo received by the probe, increasing echointensity. Indeed, in both age groups, the ultrasound penetrated the same SFT (GM 0.6 cm and VL 0.8 cm), yet echointensity was still ∼10 AU higher in older participants, which is consistent with previous findings (Akima et al., 2016). This may also explain the moderate negative correlation between echointensity and pennation angle in the current study.

Intramuscular adipose deposits in muscle are small and heterogeneous, which increases the number of reflecting interfaces. EMCL are both anisotropic and located on the perimysium and, therefore, subject to the same fibre orientation challenges that could lead to inconsistent echointensity values. Given the large capacity of muscle to accommodate heterogeneous fat versus millimetre increments in subcutaneous fat, once past a certain threshold, increases in echointensity due to fat infiltration may start to register, which has been shown in young muscular dystrophy patients (Pillen et al., 2008). Younger people, however, have been shown to have a lower SFT. With increasing age, one might expect smaller muscle thickness and increased intramuscular fat (increased echointensity) combined with thicker subcutaneous fat (decreased echointensity). The increase in subcutaneous fat as the dominant variable may cancel out any dramatic change in echointensity between middle‐aged and young. The three participants with high fat fractions are a good example of this. For these three adults, subcutaneous fat is the only variable clearly greater than in other participants (Figure 3a–c). This is reflected in higher EMCL values (Figure 2a) but not reflected in their echointensity values (Figure 2c). That these three also have the highest SFT would also explain why their raw EI is lower, with the highest SFT also having the lowest raw EI. The two older adults with lower fat fraction but very high EMCL (Figure 2a) present an interesting situation. The adult with the highest EMCL also had the lowest IMCL (0.5), the cumulative effect of which is a lower overall fat fraction. The second outlier, whilst not the second lowest IMCL value (4.4), still had an IMCL concentration well below the mean. This shift in fat content ratio (higher EMCL and lower IMCL, Table 2) could represent the inherent change as we age, but it does not occur in *all* older participants. A more plausible explanation would be the variation in physical activity level and metabolic health amongst the participants with the exercisers having a more favourable fat ratio. This is important as it affects the statistical relationship of these variables. Anecdotally, these two participants were sedentary, but as physical activity status was not collected, this cannot be generalised. Any experimental intervention that seeks to manipulate intramuscular fat (e.g., exercise training) will likely change the muscle parameters and therefore influence the echointensity value. This highlights why echointensity alone is a poor indicator of fat infiltration over time and that in its broadest sense is not a reflection of muscle fat infiltration, as previously suggested, but the resultant output of a combination of both muscle quantitative parameters and fat infiltration, which together are indicative of the health status of the muscle. It certainly lends support to the use of the multivariable regression equation that considers these variables.

The pork loin, in which muscle tissue remained constant and SFT was systematically removed, showed a clear increase in echointensity as SFT decreased in a negative exponential pattern. This negative relationship has been shown previously (Young et al., 2015) where ultrasound echointensity values were measured in five humans by applying four different levels of pressure to depress the subcutaneous fat layer over a muscle and measuring the echointensity, although it is unclear which muscle was used or whether compression of fat affects echointensity per se. An inconsistency in the derivation of the SFT correction factor presented in Young et al. (2015) was also noted. Specifically, instead of using the gradient of −39.9 (i.e., rate of change of echointensity per unit SFT) the average echointensity (40.5 AU) of the five participants at zero SFT (i.e., the *y*‐intercept) was used. This is important, because, the gradient scales with units, while the resultant echointensity does not. In the current *ex vivo* study, when log transforming the non‐linear relationship, the gradient was similar at −39.8, suggesting that although the rate of change of echointensity in the *ex vivo* model is similar to human values (Young et al., 2015), the relationship between echointensity and SFT thickness is not linear and the ‘correction’ method proposed by Young et al. (2015) should not be used. Indeed, when correcting the ultrasound fat fraction values with the correction method the ability to predict MRI fat fraction reduced by 13% and 17% in the GM and VL, respectively.

When we applied the prediction equation to ultrasound measurements from a separate cohort of 40‐ to 60‐year‐old females undergoing 12‐week resistance exercise training, there was a weak negative correlation between strength and fat fraction, which is in agreement with previous research measuring thigh fat infiltration by CT scan attenuation in older individuals (Visser et al., 2005). However, whereas resistance exercise increased muscle strength by around 20%, it did not affect fat fraction, clearly suggesting that other factors contribute to muscle strength and quality. Indeed, gains in muscle strength in older adults have been shown to be predominantly determined by neuromuscular adaptation by increasing the voluntary activation of agonist muscles (Häkkinen et al., 1998), and those greatest changes occur in the first 12 weeks (Morganti et al., 1995). It is also possible that the VL equation was unable to detect changes in fat fraction in the rectus femoris, which may have different lipid storage and muscular adaptive patterns.

Probe tilt by just 2 degrees can decrease echointensity by 4.7% and 10.5% in tibialis anterior and biceps brachaii, respectively (Dankel et al., 2020). The differences in echointensity values between groups were greater than this potential measurement error. As expected, ultrasound muscle thickness has been shown here to be a reliable and robust measure, with low variance, which is consistent with previous research (Franchi et al., 2018; Mechelli et al., 2019; Reimers et al., 1998; Sanada et al., 2006; Worsley et al., 2014). Fat fraction of the ultrasound segment was consistent with corresponding MRI segment and representative CSA, making the ultrasound region of interest a good proxy for the whole muscle. To be of further clinical use the equations would need to be validated in a separate cohort, since the current sample was not split into separate train and test sample groups for validation. These equations would also only be valid using ultrasound devices with similar settings. Focus depths can be adjusted and inter‐device reliability for muscle thickness is excellent (Ritsche et al., 2022) whereas echointensity is an acoustic property influenced by probe frequency. This study used a 7.7 Hz linear probe; these equations, therefore, would only be valid for probes with this frequency.

The validation study recruited participants under 40 years (range 21–40 years) and over 50 years (range 50–69 years), both male and female, whereas the experimental Study 3 was conducted on females aged 40–60 years. While there was nothing to suggest that the relationship between strength and fat fraction or measurement of echointensity differs between sexes, this study has shown that age can affect echointensity. Study 3 includes women aged 50–60 years, which was an age range not included in the validation study. The applicability of the model to this age group may therefore be limited.

In conclusion, we present two multivariable ultrasound equations for GM and VL, which include measures of both muscle quantity and quality that are capable of predicting fat infiltration, comparable to gold standard MRI fat fraction. At this stage the models are valid only within the parameters set in this study, approximately 2–6% fat fraction, until more data are collected in those with extreme fat percentages. The previous correction factor has been improved upon by incorporating measures of SFT, muscle thickness, echointensity and age. This low cost, accessible, easy‐to‐use bedside tool has potential to provide clinicians with valuable and immediate information on muscle quantity and fat infiltration valid for both young and middle‐aged adults. This is useful not only in the field of ageing muscle research but shows great potential for clinicians working directly with patients in a health care setting, to use as an objective marker, to assess treatment effectiveness and to direct future treatments in order to improve health outcomes.

## AUTHOR CONTRIBUTIONS

This study was conducted abroad as part of Public Health and Sport Sciences, Medical School, University of Exeter. Conception or design; acquisition, analysis or interpretation of data: all authors. Drafting of the work or revising it critically for important intellectual content: RK Holsgrove‐West, FB Stephens. BT Wall. PM Słowiński. R Revuelta Iniesta. J Fulford. Funding acquisition: RK Holsgrove‐West and FB Stephens. Supervision: FB Stephens. BT Wall. and R Revuelta Iniesta All authors approved the final version of the manuscript and agree to be accountable for all aspects of the work in ensuring that questions related to the accuracy or integrity of any part of the work are appropriately investigated and resolved. All persons designated as authors qualify for authorship, and all those who qualify for authorship are listed.

## CONFLICT OF INTEREST

None declared.

## Data Availability

Data can be made available upon reasonable request by contacting f.b.stephens@exeter.ac.uk.
